# Psychosocial impacts of training to provide professional help: Harm and growth

**DOI:** 10.1177/1460408620968340

**Published:** 2022-04

**Authors:** Jacqueline Ball, Clare Watsford, Brett Scholz

**Affiliations:** 1 Faculty of Health, University of Canberra, Australian Capital Territory, Australia.; 2 ANU Medical School, Australian National University, Australian Capital Territory, Australia

**Keywords:** Vicarious trauma, moral injury, compassion fatigue, secondary traumatic stress, burnout, vicarious posttraumatic growth, helping professionals, trainee helping professionals, psychologists, trainee psychologists

## Abstract

**Introduction:**

Research has consistently demonstrated professionals in helping roles (“helping professionals”) experience vicarious trauma, moral injury, compassion fatigue, secondary traumatic stress, and burnout. Vicarious post-traumatic growth has also been identified in the literature. This article aimed to contribute to understanding the experiences of these constructs of trainee helping professionals. Emphasis was placed on how to foster vicarious post-traumatic growth.

**Methods:**

A qualitative semi-structured interview was designed to enable the researchers to explore the experiences of 14 trainee psychologists from an Australian Master of Clinical Psychology program.

**Results:**

It was identified that burnout, and beginning stages of vicarious trauma, moral injury, compassion fatigue, and secondary traumatic stress might occur during psychologists’ training. Five elements underpin vicarious post-traumatic growth, four of which were reflected in this article. A need and suggestions for how to further develop vicarious post-traumatic growth are discussed.

**Conclusion:**

This research could go on to be applied to curriculum development and practice policy, ultimately leading to improved early-intervention and ongoing systems of support for helping professionals. This, in turn, would improve quality of care in communities.

## Introduction

Throughout human history, innumerable professional and non-professional roles have existed and will exist to provide a form of care, or help,^1^ to members of all communities across the globe, in countless contexts. Irrespective of the role or context, one constant is that the helping process significantly influences both recipients of help and the helpers.^2^ Much of the research on the helping process has focused on recipients’ responses, although there is now growing research focused on the helper.^2^

Previous research on how “helping professionals” have responded to the process of providing professional help has identified a number of constructs. The language used in the literature has suggested that these constructs exist along a spectrum of negative (or harmful), neutral, and positive responses,^3^ which are not necessarily mutually exclusive. However, the wellbeing of helping professionals, their organisations, and the outcomes of those they are providing care to^4^ rely on identification of ways to attend to responses that are harmful and achieve and maintain positive responses.

Much of the research on how established helping professionals are impacted by the process of their work has focused on harmful responses, of which this article focuses on five related but distinct constructs: vicarious trauma (VT),^5^ moral injury (MI),^6^ compassion fatigue (CF),^7^ secondary traumatic stress (STS),^8^ and burnout.^9^ VT involves a disruption in a person’s sense of safety, trust, esteem, intimacy, and control,^5^ resulting in negative perception of one’s self, others, and the world.^10^ VT can occur when helpers are empathically engaged with the traumatic experiences of those they are providing help to, usually through cumulative trauma-related helping experiences.^11^ VT is based on the constructivist self-development theory, which considers that an individual’s previous life experiences are likely to determine the impact future traumatic experiences have on that individual.^5^

MI can occur when a person’s moral values are violated as a result of traumatic events,^6^ resulting in significant distress and impairments to functionality, caused by self-blame and shame, and disruptions to trust and spirituality/existentialism.^6^ Research on MI originated with, and has mostly focused on, military veterans, but is increasingly being applied to a wider context. It has been recommended that MI be understood and addressed within the framework of a biopsychosocial-spiritual model, in which people need to be considered as whole persons: their physical, psychological, social, and spiritual parts all need to be considered for their needs to be met.^12^

The other three harmful constructs; CF, STS, and burnout are related to a stress-process framework, which considers that in order to process a stressor, physiological as well as psychological responses are required of the individual.^13^ CF refers to emotional and physical exhaustion through providing compassion,^14^ and like VT, occurs through cumulative helping experiences. CF leads to gradual desensitisation to the experiences of those being helped, which can result in an increase in professional errors, higher rates of detrimental effects on mental health, and higher rates of leave taken due to being stressed. In turn, this can lead to decreased quality of care for people in need of help, poor workplace conditions, and negative impacts on personal life.^14^

STS occurs through secondary experiences of traumatic content.^15^ STS can be the result of being exposed to a single second-hand traumatic experience or cumulative trauma-related helping experiences, and results in explicit symptoms that resemble those of primary exposure to traumatic stress.^16^ These symptoms include intrusive imagery, avoidance of reminders and cues, hypervigilance, exhaustion, and numbing,^8^ and at times warrant a diagnosis of Post-traumatic Stress Disorder (PTSD).

Burnout involves chronic exposure to environments with high levels of stress.^17^ Sources of stress can be exposure to trauma, fear or uncertainty, loss of economic security or position, and lack of control over circumstances.^18^ Burnout results in gradual loss of optimism, energy, and goals; and on a greater scale, alienation, dissatisfaction, and ultimately departure from a workplace. This state of emotional and mental exhaustion creates physiological consequences including fatigue, irritability, and physical complaints, and can also lead to personal problems such as negative self-esteem, poor attitude, and reduced efficiency and effectiveness.^18^

To demonstrate how these five constructs interact with each other, consider a fictional paramedic in New York City in the midst of COVID-19. This paramedic has had an indescribable increase in and change to workload, had colleagues who lost their lives to COVID-19, and has not been able to go home to sleep or be with loved ones due to risk of potentially spreading COVID-19 to them - all on top of a job that was already taxing prior to COVID-19. Witnessing so many patients of critical illness and death could lead to STS and VT (although it is likely there are elements of primary, as well as secondary or vicarious traumatic stress in this example). Deciding which call outs and subsequently which patients’ lives to prioritise could lead to MI, as could wondering why America’s leaders did not act differently in response to the COVID-19 pandemic. Providing compassion to patient after patient could lead to CF. Finally, such high levels of stress, fear and uncertainty, and lack of control, could lead to an experience of burnout for the paramedic. This example demonstrates that while these five constructs appear to overlap in ways, it is important to distinguish them. Correct identification is essential in addressing their occurrence.

While a vast amount of research has examined harmful responses experienced by helping professionals, research on responses that are positive is underdeveloped.^19^ An early study that directly explored positive responses of trauma work sampled 21 metropolitan psychotherapists, who on average had 16.9 years of clinical experience, and a caseload consisting of 45% “trauma work”.^2^ This research specifically focused in depth on the “positive” and “negative” responses these psychotherapists experienced through their work, by utilising a semi-structured interview that asked “How have you been affected by your work with clients who have experienced traumatic events?” All participants reported experiences constituting what the authors coined vicarious post-traumatic growth (VPTG), described as growth following vicarious exposure to trauma. The authors recommended further research on VPTG.

Subsequent research has focused on vicarious experiences of traumatic events such as war, torture, the Holocaust, domestic violence, sexual assault, the 2001 “9/11” terrorist attacks, refugee assistance, and funeral assistance.^20^ The research on VPTG has demonstrated that the growth seen in those who vicariously experienced trauma reflected the five aspects of growth that underpin Tedeschi and Calhoun’s “posttraumatic growth” (PTG) which describes the phenomenon of how experiences of direct trauma can elicit growth,^21^ on which VPTG was based - both relating to the constructivist self-development theory.^5^ The five aspects of growth seen in PTG, reflected in VPTG, include: improvements in interpersonal relationships, a greater appreciation for life, new opportunities or pathways in life, a greater sense of personal strength in ability to cope with crises, and spiritual changes or development.^21^ In 2015 Manning, Terte, and Stephens^20^ conducted a review of factors that are facilitative of VPTG which can be reviewed online in Supplementary File 1.

Research regarding the helping process has focused on established helping professionals more than trainees with few studies describing positive changes, growth, and PTG experienced by trainee helping professionals,^22–24^ but none regarding positive responses related specifically to VPTG. It seems that the research that exists on harmful responses experienced by trainees is also limited and does not adequately explore how VT, MI, CF, STS, and burnout might apply; furthermore, existing research indicates that trainees require unique forms of support, but that such support is not consistently designed and provided.^25,26^ Qualitative sampling of physiotherapy trainees on their experiences of management of patient death^27^ disclosed that during their student placements, they experienced established helping professionals to be “insensitive” and “blasé” in response to patient death, which in turn disallowed trainees to be open and transparent about, and subsequently process, their own emotional distress. In order to provide consistent, informed support for trainees in helping professions, their experiences of the process of providing help needs to be better understood; this understanding would enhance wellbeing and development for helping professionals during their training and subsequently as their careers evolve over time, which in turn, would improve quality of care in communities.

## Methods

The aims of this research were to collate data obtained from trainee clinical psychologists in their final year of an Australian Master of Clinical Psychology (MCP) program, with specific regard to their experiences of their clinical work and to analyse these data with regard to positive and harmful ways trainees have been impacted by their clinical work.

### Design

A cross-sectional, semi-structured, individual interview design was utilised. Open-ended questions were used to enable interviewees to provide information most important and relevant to their experiences,^28^ and for individual experiences to be analysed in depth.^29^ Given the need for development of the research on trainees in helping professions, this technique was advantageous as it allowed opportunity for participants to share their most significant experiences.

Participants were asked to identify their age, gender, previous clinical experience prior to commencing their MCP degree, and whether they had general registration as a psychologist. In Australia, “general registration” qualifies an individual as a psychologist, albeit not a clinical psychologist, and, at times, psychologists with general registration return to university to obtain clinical qualification. It was important to consider the influence of general registration or any other previous clinical experience on interviewees’ responses.

### Interview questions

A set of open-ended interview questions was developed targeting the constructs identified in the literature relating to participants’ experiences of their clinical work. The researchers developed more specific questions for each of the open-ended questions to prompt participants who required help to understand the types of experiences they might talk about. Questions targeting participants’ self-care knowledge and engagement were also included in the interview schedule. Self-care can be implemented by helping professionals to protect against harmful experiences such as VT, MI, CF, STS, and burnout and is defined by the World Health Organisation as individuals having the ability, as active agents, to promote health, prevent disease, maintain health, and cope with illness and disability, for themselves, with or without the support of a health worker.^30^ Data obtained through the self-care questions that were informative in direct relation to harmful and positive responses experienced by trainee psychologists have been included in the results section. Questions and prompts relating to reflective-practice and participants’ understanding of VT, CF, and PTG were also included in the interview schedule. However, data obtained relating to reflective-practice and understanding of VT, CF, and PTG were incomplete and therefore not analysed.

The full schedule of interview questions and complete version of results is available online in Supplementary File 2.

### Research setting and participants

A purposive sampling technique was used to recruit students in their final year of the two year, full-time MCP at the University of Canberra (UC), a mid-sized, regional Australian university. All enrolled students were required to be provisionally registered as a psychologist with the Australian Health Practitioner Regulation Agency (AHPRA). An introduction to working with clients presenting with trauma was provided, however the training focus was on cognitive behavioural therapy. First year students participated in an internal placement at UC’s clinic, in which assigned client presentations were assessed to be mild (typically anxiety or mood disorders), however, in some cases, clients were deemed to have had “trauma experiences”. For second year students, placements were external to UC and involved greater exposure to clients who had experienced trauma. All organisations that accepted students for placement were located in Canberra, Australian Capital Territory. Examples include: Child and Adolescent Mental Health Service, Adult Mental Health Service, forensic settings, Perinatal Mental Health, and the Crisis Assessment and Treatment Team. Any student who worked with a “trauma presentation” received specialist supervision from an experienced clinical psychologist.

Fourteen of the 41 enrolled second year students (34.2%) agreed to participate, who were predominantly female (11/14; 78.6%) with three males (21.4%) across the ages of 23-55 years (mean 31.21, *SD* 10.18); two participants were enrolled as part-time students. There were no reports of prior clinical experience nor of general registration. The sample profile (age, gender, enrolment-type, and previous experience) was reflective of UC’s MCP program second year cohort as a whole.

### Data collection

The University of Canberra’s Human Research and Ethics Committee approved the study design before commencement. Participants were recruited via student online forums and an in-class announcement and interested students were emailed an information and consent form, which contained an overview of the nature and purpose of the study. Students then confirmed their participation, by sending a response email to arrange a date and time for an interview.

Before each interview commenced, a hardcopy version of the information and consent form was read, clarified, and signed by participants, demographic information was collected and verbal consent to an audio-recorded interview obtained. The interviews ranged from 19:24 to 64:35 minutes (mean 47:51, *SD* 12:92). At the end of each interview participants were given a movie ticket voucher to thank them for their participation in the study. All participants accepted the offer to be sent a summary of the results.

The semi-structured interviews were conducted in a private room on UC campus. The year they took place has been omitted to protect participant confidentiality. The first two interviews were co-run by the principle researcher and an independent researcher who had a background of qualitative research and interviewing experience. The independent researcher was a member of staff from UC’s Faculty of Health, but from the nursing rather than psychology discipline, and therefore not affiliated with the participants derived from the MCP program. The third interview was run by the principal researcher, with the observation of the independent researcher, to ensure consistency in interviewer approach. The principle researcher solitarily conducted the remaining 11 interviews. Recruitment continued until the sample was reflective of UC’s MCP program second year cohort as a whole, and saturation of data occurred. Participants were given the opportunity to review the accuracy of their interview transcripts. One participant took up this option but requested no changes.

### Data analysis

Data were transcribed verbatim, and later coded in NVivo 11 qualitative data analysis software (QSR International Pty Ltd., 2015) using thematic analysis. The guidelines on thematic analysis formulated by Braun and Clarke^31^ were followed to achieve systematic identification, conduct analysis, and compose reports on patterns of participants’ experiences within the data. The data-driven themes and sub-themes were either determined through identifying meaningful surface content or meaning underlying the surface content from the interview transcripts.^31^ Patterns found across and within the 14 interview transcripts were organised into initial overarching themes^32^ and then reanalysed until these themes and their sub-themes were established with meaningful descriptions and labels.^33^

To indicate the frequency of participant responses, four levels were used to report sub-themes. A *rare* sub-theme was one that was reported by two participants, *variant* by more than two but less than half, *typical* by half or more, and *general* by all or all but one participant.^34^ Data reported by one participant only were not included.

## Results

**Figure 1.**
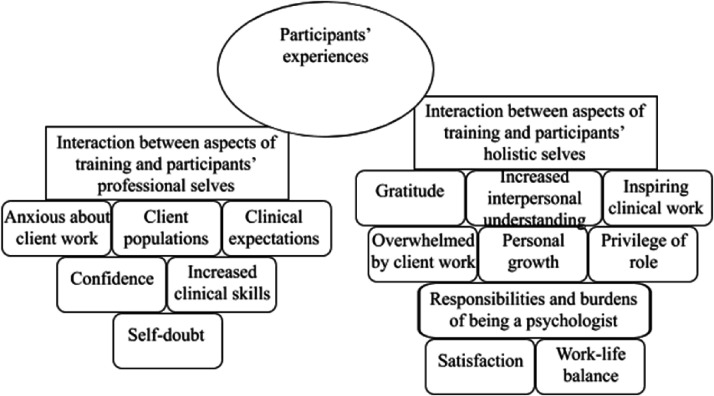
Summary of the sub-themes relating to participants’ experiences.

Two overarching themes contributed to this results section, both of which contained several sub-themes. The first theme (Figure 1) was *participants’ experiences* and contained two sub-themes: *interaction between aspects of training and participants’ professional selves*, which contained six further sub-themes (anxious about client work, client populations, clinical expectations, confidence, increased clinical skills, and self-doubt) *and interaction between aspects of training and participants’ holistic selves*, which consisted of nine further sub-themes (gratitude, increased interpersonal understanding, inspiring clinical work, overwhelmed by client work, personal growth, privilege of role, responsibilities and burdens of being a psychologist, satisfaction, and work-life balance).

**Figure 2.**
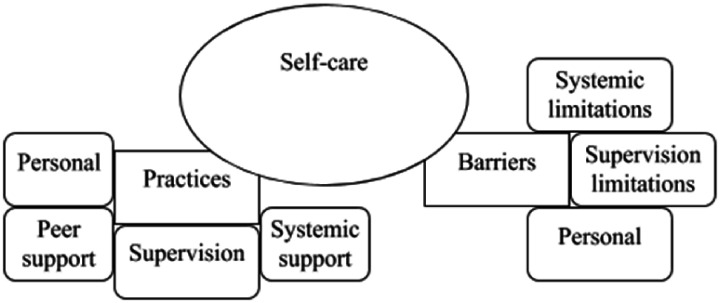
Summary of the sub-themes relating to self-care.

The second theme (Figure 2) was *self-care* and contained four sub-themes including knowledge, practices, frequency, and barriers. Knowledge and frequency were not included in this results section as they did not fit the purpose of this article. The authors are contactable for access to the full self-care analysis. *Practices* consisted of four further sub-themes: personal, peer support, supervision, and systemic support. *Barriers* consisted of three further sub-themes: personal, supervision limitations, and systemic limitations.

A more detailed version of these results can be found under Results in online Supplementary File 2: Interview Questions and Complete Version of Results.

## Discussion

This study sought to explore trainee psychologists’ experiences of their clinical training with a focus on how these experiences relate to the constructs of VT, MI, CF, STS, burnout, and VPTG; and that they might have developed over the course of training. Self-care results have been discussed as they relate to these same constructs; additional experiences reported by the trainee population are also noted.

### Indications of VT, MI, CF, STS, and burnout

Of concern, markers of burnout identified in the literature appeared present within trainee psychologists, seen in the sub-themes “overwhelmed by client work” and “work-life balance”, as well as in the self-care sub-themes “supervision limitations” and “systemic limitations”. Indicators of VT, MI, CF, and STS were not present in trainee psychologists’ experiences. However, the “client populations” sub-theme reveals that participants reported being most challenged by clients who were suicidal or had experienced abuse or trauma; exposure to such client groups could lead to experiences of VT, MI, and STS. In addition, the sub-theme “responsibilities and burdens of being a psychologist” could be an early marker of CF. These results indicate that the beginning stages of the harmful constructs identified in literature might occur as early as the training phase of a career as a psychologist.

### Indications of VPTG

Similarities were found between the results of the study reported in this article and four of the five aspects of growth that were originally seen in Tedeschi and Calhoun’s work on PTG^21^ and later reflected in research on VPTG.^2^ The sub-theme “increased interpersonal understanding” is closely aligned with Tedeschi and Calhoun’s improvements in interpersonal relationships, “gratitude” can be compared to having a greater appreciation for life, “inspiring clinical work” relates to new opportunities or pathways in life, and similarities can be drawn between “personal growth” and having a greater sense of personal strength in ability to cope with crises. However, it is important to note that although “inspiring clinical work” relates to new opportunities or pathways in life, trainee psychologists spoke of this sub-theme in terms of the experiences of humankind in general as opposed to themselves directly. Previous literature^2,32^ (Supplementary File 1) has also highlighted that participants viewed development of personal strength as a phenomenon, and spiritual growth as a belief, experienced by humankind rather than themselves as individuals. This might further confirm a distinction between PTG and VPTG.

Tedeschi and Calhoun’s^21^ spiritual changes or development was not reflected in the trainee psychologists. In the “personal” self-care section of the results, only two participants spoke of spiritual affiliations. Perhaps then, an absence of spiritual experience within this group of trainee psychologists was the reason these results did not emerge. However another plausible explanation is that participants might not have been inclined to spontaneously talk about this part of their lives or selves.

The results of the analysis obtained from the trainee psychologist sample indicated similarities to five of 13 factors that Manning et al. identified to facilitate VPTG (see Supplementary File 1).^20^

### Additional experiences

Trainee psychologists experienced some phenomena that do not relate to the literature on VT, MI, CF, STS, Burnout, and VPTG introduced in this article. These phenomena were noted through the sub-themes “anxious about client work”, “clinical expectations”, “confidence”, “increased clinical skills”, “satisfaction”, “self-doubt”, and “privilege of role”. The existence of these sub-themes could be explained by the trainee psychologist’s focus on integrating new learning. Additionally, these sub-themes might further confirm what prior research^35^ (Supplementary File 1) has indicated regarding factors that make VPTG distinct from PTG; desire to make a difference in order to obtain a sense of meaning.

### Strengths and limitations

This study that explores positive responses directly related to VPTG experienced by trainee helping professionals is unique in the literature and also contributes unique research on trainees’ experiences of harmful responses. The prospective effects of this research could enhance the personal and professional experiences of trainee helping professionals, and subsequently established helping professionals, in turn improving delivery of care to communities.

It is likely that inclusion of self-care literature alongside the entirety of the self-care results and an interpretation of those results would have provided valuable implications and future research suggestions; similarly, focus on the concept of reflective-practice and participants’ understanding of VT, MI, CF, STS, burnout, and VPTG would have been worthwhile but the interviewing process did not sufficiently focus on obtaining these data. Further, trainees require unique forms of support and that support is not consistently designed and provided,^25–27^ and whilst the interview question “How have you found the Master of Clinical Psychology program has supported you throughout your experience of training to become a clinical psychologist?” led to data that spoke to this concern, it would have been useful to intentionally include an interview question that directly explored what participants believed they need.

#### The definition of VPTG

Perhaps the greatest limitation of this research is that one of the constructs it centres on, VPTG, currently lacks a clear definition. The human experiences that VPTG has been applied to within research contexts to date^20^ are all quite extreme; as can be argued is the clinical definition of psychological traumatic stress.^36,37^ The only definition of trauma and traumatic stress in the current version of the Diagnostic and Statistical Manual of Mental Disorders (DSM-5) is found within the section on PTSD. This DSM-5 definition of traumatic stress requires “actual or threatened death, serious injury, or sexual violence”.^37^ In this definition, experiences related to stressful events that do not involve an immediate threat to life or the physical body (such as cancer or physical neglect), and psychosocial stressors (such as emotional abuse, the breakdown of a relationship, or job loss) cannot be considered trauma.^37^ The definition of “post-traumatic growth” in VPTG is not as clear as that for “post-traumatic stress” in PTSD. However, the five aspects of growth that underlie VPTG are clear.^21^

Does a clinically significant definition for psychological traumatic stress have to be met for phenomena, such as the five aspects of growth outlined in the VPTG construct, to occur on individual and collective levels throughout humanity? In the words of one participant from the study reported in this article:

*… they come with their struggles and I see … a sense of humanity … we're all people, we all struggle … it makes me reflect on my own humanity …*

This quote conveys that struggle, or suffering, is common among all people, no matter their circumstance - it is all around us on an everyday basis. This, however, is not the standout commonality shared by the five harmful constructs of VT, MI, CF, STS, and burnout - indeed, the greatest thing that stands out within these constructs is disconnection. Disconnection to others, oneself, and life itself in all five constructs, and to morality in MI, compassion in CF, and the workplace in burnout.

To the contrary, connection is the stand out commonality shared for the five aspects of growth in VPTG.^21^ Improvements in interpersonal relationships, a greater appreciation for life, new opportunities or pathways in life, a greater sense of personal strength in ability to cope with crises, and spiritual changes or development. Connection to others, oneself, life itself, and even struggle or suffering, lives within VPTG.

Akin to the concern expressed^36^ by critics regarding the DSM-5 definition of a traumatic stressor, the authors ponder whether it is responsible to place heavily weighted emphasis on the cause of growth as well as the growth itself. Perhaps this clinical question can only be answered by first considering some philosophical questions. Are we as humans inborn with the capacity for such growth, but it is sometimes dormant, and not always naturally activated? Is something world-wide like COVID-19, with the spectre of irreversibility like global warming has the potential to become,^38^ necessary for activation of widespread lasting connection? That would be ironic. How can activation be fostered without extreme harm having to occur? Is it possible for it to be activated in people who are “without a conscience”? The authors suggest it might be up to those of us with a conscience to implement further research in attempts to find out the answers to questions like these.

### Implications for training helping professionals and future research

Four of Tedeschi and Calhoun’s five components of VPTG^21^ are reflected in data provided by this research, which supports that VPTG might occur within trainee helping professional populations. If similar results were found in further exploratory work that replicated this study but sampled different trainee helping professions, this finding of VPTG occurring in trainee populations could be stated with more confidence. One of these five factors was not indicated in the findings of this study - “spiritual changes or development”. Future research could implement a prompt to specifically explore experiences of “spiritual changes or development” within trainees’ helping experiences. If this is a more prevalent experience than this study indicated, and the reason it was not indicated is participants’ non-inclination to spontaneously share this aspect of their lives or selves, asking a sensitively designed prompt could encourage disclosure for this particular component of the VPTG construct.

The findings of this study might act as a gateway for obtaining further insight regarding the mechanisms that lead to VPTG which is explored further in Supplementary File 1. More exploratory research of a multidisciplinary nature, would provide a more reliable indication of the occurrence of harmful impacts on trainees, i.e. if experiences of burnout do occur, and if there are potential early markers of VT, MI, CF, and STS at the training stage of a helping career. Furthermore, application of a longitudinal method obtaining data from helping professionals at different phases of their careers, including the trainee, early career, and later career phases, might provide insight on how the experiences of these professionals develop over time. Any research identifying the occurrence of VT, MI, CF, STS, burnout, and VPTG in the training phase of a career as a helping professional would aid in developing strategies to counteract or transform harmful and foster positive responses. It would also be useful if future research investigated the design of existing training programs, with both a trauma- and non-trauma-specific training focus, from a variety of helping disciplines, to determine how training professionals are already being supported or need to be better supported according to their unique experiences and needs.

The authors believe this research could be used to strategically tailor early-intervention and ongoing systems of support which is a significant implication for the field. If applied to curriculum development for training programs and practice policy for workplaces, far-reaching improvements to helping professionals’ experiences could be achieved.

## Conclusion

The authors recommend that initial future research should be explorative and focus on determining whether these results are reliable. If it is the case that VT, MI, CF, STS, and burnout and the five VPTG components and 13 VPTG factors do apply to multidisciplinary trainee as well as established helping professionals, there might be a relationship between the ways trainee and established helping professionals respond to their work. Clarifying whether this relationship does exist would improve methods to address each respective group’s experience.

This study created a foundation from which the trainee helping professional research can be expanded, which would have highly beneficial consequences, with the resultant knowledge informing education and training resources, in turn contributing to support of the professional and personal growth of trainee helping professionals across disciplines. This in turn, might shed light on experiences of established helping professionals further along in their careers, and how they can be supported. Ultimately delivery of care provided to communities would be enhanced. The exploratory research presented in this paper reminds us all that practitioners in the helping professions should be protected all stages of their careers.

## Supplemental Material

sj-pdf-1-tra-10.1177_1460408620968340 - Supplemental material for Psychosocial impacts of training to provide professional help: Harm and growth Click here for additional data file. Supplemental material, sj-pdf-1-tra-10.1177_1460408620968340 for Psychosocial impacts of training to provide professional help: Harm and growth by Jacqueline Ball, Clare Watsford and Brett Scholz in Trauma

sj-pdf-2-tra-10.1177_1460408620968340 - Supplemental material for Psychosocial impacts of training to provide professional help: Harm and growth Click here for additional data file. Supplemental material, sj-pdf-2-tra-10.1177_1460408620968340 for Psychosocial impacts of training to provide professional help: Harm and growth by Jacqueline Ball, Clare Watsford and Brett Scholz in Trauma
