# Supplementary material for: Psychosocial impacts of training to provide professional help: Harm and growth
Source: Trauma. 2022 Apr;24(2):115–23. doi: 10.1177/1460408620968340 (PMC7642821; doi:10.1177/1460408620968340)
Supplement: sj-pdf-1-tra-10.1177_1460408620968340 - Supplemental material for Psychosocial impacts of training to provide professional help: Harm and growth [file sj-pdf-1-tra-10.1177_1460408620968340.pdf]

Fostering Vicarious Posttraumatic Growth (VPTG)

How Can VPTG be Fostered?

To facilitate the experience of VPTG, it is important to explore how it can be facilitated. In 2015 Manning, Terte, and Stephens(20) conducted what they claim to be the first, and appears to currently be the only comprehensive review of factors facilitative of VPTG. In their work, Manning et al. identified thirteen factors, categorised as cognitive and psychological, behavioural, interpersonal, or external (see Table 1).

Table 1

Overview of the 13 factors identified as facilitative of VPTG and explanation of why the factors are facilitative

Category	Factor	Explanation
Cognitive and Psychological	Empathic engagement(22)	Hypothetical application of others' trauma to one's own life(23)
	Humanistic and transpersonal theoretical orientation(23) (Relevant only to therapists)	Therapeutic philosophy that suffering provides opportunities for growth and development(23)
	Professional self-esteem(24)	Sense of satisfaction, competence, and value in one's work(25)
	Negative affect(26)	VPTG must be preceded by experiences of devastation and distress following vicarious exposure to trauma(27)
	Optimism and positive affect(28)	Identification of the positives that can come from suffering(29)
	Resilience(30)	Allows coping in face of adversity, however, reduction of impact of trauma may also hinder VPTG(20)
	Sense of coherence(31)	Viewing the world as comprehensible, manageable, and meaningful(31)
Behavioural	Self-care(2)	Reduce distress and protect wellbeing(18)
	Therapy(29)	Allows opportunity to process vicarious exposure to trauma(32)
Interpersonal	Social support(29)	From peers and supervisors, but not the organisation in which the helper works or family and friends(29)
	Witnessing PTG(2)	Inspiration through witnessing the strength of humankind(33)
External	Time(33)	Allows opportunity to process and find meaning in vicarious exposure to trauma(33)
	Trauma history(34)	Familiarity with processing of traumatic material(35)

The studies on VPTG that identified the 13 factors overviewed in Table 1 were conducted through a mixture of qualitative and quantitative methods. The quantitative methods used a measure designed for PTG, as there is currently no known measure for VPTG. Although VPTG and PTG have been shown to have remarkable similarities, the review conducted by Manning et al. showed that the two constructs also have significant differences.(20) Differences include individuals who had experienced VPTG regarding "personal strength" as a quality of humankind rather than applying it to themselves as individuals(2,32), and shifts in outlook on "spiritual growth" as a healing tool rather than a personal belief.(2) In addition, a unique aspect to VPTG was identified; meaning derived through being a professional who makes a difference.(36) This is indicative that further research is required to operationalise and measure, and ultimately facilitate, VPTG.

Indications of the 13 VPTG Factors in the Study Reported in this Article

Sub-themes matched five of the 13 factors demonstrated as underlying VPTG(20), which were displayed in Table 1. Elements of the sub-themes "clinical expectations", "gratitude", "increased interpersonal understanding", "inspiring clinical work", "personal growth", and "responsibilities and burdens of being a psychologist" relate to empathic engagement.(22,23) Elements of "anxious about client work", "confidence", "increased clinical skills", "privilege of role", "satisfaction", and "self-doubt" relate to professional self-esteem.(24,25) Furthermore, elements of "inspiring clinical work" and "satisfaction" relate to witnessing PTG.(2,33) Forms of "peer support", "personal" support, "supervisor support", and "systemic support" all relate to social support.(29) However, a difference exists between social support in the study reported in this article and previous research, in that previous research identified support from peers and supervisors, but not support from the organisation in which the helper works or family and friends, as facilitative of VPTG.(29) Sub-themes relating to peer, supervisor, systemic, and personal forms of social support were identified,

with trainee psychologists believing they were supported in all four forms. In addition, comparable forms of self-care were seen in both previous literature(2,18) and the study reported in this article.

Data obtained from the trainee psychologists did not reflect the remaining eight factors identified as facilitative of VPTG in earlier literature. Six of these remaining eight factors involve the helper embodying negative affect(26), optimism and positive affect(28), sense of coherence(31), resilience(30), having their own trauma history(34), and engaging in their own therapy.(29) It makes sense that information relating to the latter two of these five factors, participants' own trauma background and engagement in therapy, might not have been divulged given the sensitivity of the content. Regarding experiences of negative affect, possession of an optimistic disposition, sense of coherence, and resilience, perhaps targeted quantitative measurement is required to obtain data.

The final two factors are time(33) and whether trainee psychologists employ humanistic and transpersonal theoretical orientations.(23) Data relating to application of humanistic and transpersonal theoretical orientations did not come forth from our interviewees, perhaps because it might not be a conscious, or intentional focus at the trainee psychologist level. The seventh factor, time, relates to the interview questions designed to explore changes in experiences over time. Data regarding changes over time emerged for two sub-themes, with trainee psychologists reporting that being "anxious about client work" dissipated with time, and the "clinical expectations" they held shifted with time. These results might indicate that if the constructs VT, MI, CF, STS, and burnout, as well as VPTG exist within trainee helping professional populations, a greater period of time must pass in order for them to progress.

Implications of Indications of the 13 VPTG Factors in the Study Reported in this Article

The authors suggest that the research findings of the study reported in this article might act as a gateway for obtaining further insight regarding the mechanisms that lead to VPTG. The results of the analysis obtained from the trainee psychologist sample indicated similarities to five of the 13 factors that Manning et al.(20) identified facilitate VPTG. More similarities might have been identified had alternate forms of measurement been implemented for the eight factors that were not indicated. Similar to the suggestion made for Tedeschi and Calhoun's(21) "spiritual changes or development", further exploratory research on trainee helping professionals could implement specifically targeted and sensitively designed prompts to measure the following seven of the aforementioned eight factors: trainee psychologists having "negative affect"(26), "optimism and positive affect"(28), "sense of coherence"(31), "resilience"(e.g. 30), their own "trauma history"(e.g. 34), engagement in their own "therapy"(29), and whether they employ "humanistic and transpersonal theoretical orientations".(23) For example, participants could be specifically asked if they think they are aligned with humanistic and transpersonal theoretical orientations, or believe they integrate it into their practice. While "humanistic and transpersonal theoretical orientations" was only relevant to therapists in previous research, as shown in the work of Manning et al.(20), the philosophy underpinning humanistic and transpersonal therapeutic orientations could be extended to other types of helping professionals. With respects to the eighth factor that was not indicated in this study, "time"(33), addition of longitudinal research in the future would be advantageous. The authors suggest that if further insight regarding the mechanisms that lead to VPTG was established, potentially, development of a quantitative measure for VPTG might be enabled, which in turn would allow for richer data to be obtained, ultimately expanding the understanding of VPTG.

References

39. Brockhouse R, Msetfi RM, Cohen K, et al. Vicarious exposure to trauma and growth in therapists: the moderating effects of sense of coherence, organizational support, and empathy. *J Traum Stress* 2011; 41: 735–742.
40. Linley PA and Joseph S. Therapy work and therapists' positive and negative well-being. *J Soc Clin Psychol* 2007; 43: 385–403.
41. Carmel S, The professional self-esteem of physicians scale, structure, properties and the relationship to work outcomes and life satisfaction. *Psychol Rep* 1997; 80: 591–602.
42. Taubman-Ben-Ari O and Weintroub A. Meaning in life and personal growth among pediatric physicians and nurses. *Death Stud* 2008; 49: 621–645.
43. Linley PA and Joseph S. Positive and negative changes following occupational death exposure. *J Trauma Stress* 2005; 18: 751–758.
44. Davis CG and Macdonald SL. Threat appraisals, distress and the development of positive life changes after September 11th in a Canadian sample. *Cogn Behav Ther* 2004; 50: 68–78.
45. Shiri S, Wexler ID and Kreitler S. Cognitive orientation is predictive of posttraumatic growth after secondary exposure to trauma. *Traumatology* 2010; 16: 42–48.
46. Taku K. Relationships among perceived psychological growth, resilience and burnout in physicians. *Pers Individ Dif* 2014; 59: 120–140.
47. Linley PA, Joseph S and Loumidis K. Trauma work, sense of coherence, and positive and negative changes in therapists. *Psychother Psychosom* 2005; 74: 185–188.
48. Splevins KA, Cohen K, Joseph S, et al. Vicarious posttraumatic growth among interpreters. *Qual Health Res* 2010; 20: 1705–1716.
49. Pennebaker JW, Kiecolt-Glaser JK and Glaser R. Disclosure of traumas and immune function: health implications for psychotherapy. *J Consult Clin Psychol* 1988; 56: 409–415.
50. Kjellenberg E, Nilsson F, Daukantaitė D, et al. Transformative narratives: the impact of working with war and torture survivors. *Psychol Trauma* 2014; 6: 120–145.
51. Schaefer JA and Moos RH. In: Tedeschi RG, Park CL and Calhoun LG (eds) *Posttraumatic growth: positive changes in the aftermath of crisis.* Mahwah, NJ: Lawrence Erlbaum Associates Publishers, 1998, pp.99–142.
