# Supplementary material for: Psychosocial impacts of training to provide professional help: Harm and growth
Source: Trauma. 2022 Apr;24(2):115–23. doi: 10.1177/1460408620968340 (PMC7642821; doi:10.1177/1460408620968340)
Supplement: sj-pdf-2-tra-10.1177_1460408620968340 - Supplemental material for Psychosocial impacts of training to provide professional help: Harm and growth [file sj-pdf-2-tra-10.1177_1460408620968340.pdf]

Interview Questions and Complete Version of Results

Interview Questions

A set of open-ended interview questions was developed targeting the constructs identified in the literature. The questions related to participants' experiences of their clinical work. The researchers developed more specific questions for each of the open-ended questions to prompt participants who required help to understand the types of experiences they might talk about.

The open-ended questions and prompts were: "Generally speaking, how do you think the experience of training to become a psychologist has changed you personally? (Prompt: Are there negative/positive changes you have noticed?)", "How has your experience of seeing clients changed you personally? (Prompt: What are the negative/positive changes you have noticed in yourself and your experiences?)", "What have been the most challenging/inspiring aspects of your experience in clinical placements?", and "How have those experiences changed during the course of your degree so far? (Prompts: Plot timeline on how experiences have changed over time, particularly over the three placements i.e. beginning, middle, end of first year; beginning of second year and now. Focus on anxiety levels, confidence, challenges, strengths, and personal growth at these distinct time points.)"

The questions relating to self-care were: "What do you know about self-care?", "What is your personal experience of engaging in self-care?", "What kind/s of self-care do you engage in?", "How often do you engage in those acts of self-care?", "Does that frequency feel enough/too much/not enough?", "Are there any particular barriers to engaging in regular self-care?", and "How have you found the Master of Clinical Psychology program has supported you throughout your experience of training to become a clinical psychologist?"

Results

Two overarching themes contributed to this results section. Both contained several sub-themes. The first theme (see Figure 1) was *participants' experiences* and contained two sub-themes. The first sub-theme was *interaction between aspects of training and participants' professional selves*, which contained six further sub-themes: *anxious about client work, client populations, clinical expectations, confidence, increased clinical skills,* and *self-doubt*. The second sub-theme was *interaction between aspects of training and participants' holistic selves*, which consisted of nine further sub-themes: *gratitude, increased interpersonal understanding, inspiring clinical work, overwhelmed by client work, personal growth, privilege of role, responsibilities and burdens of being a psychologist, satisfaction,* and *work-life balance*.

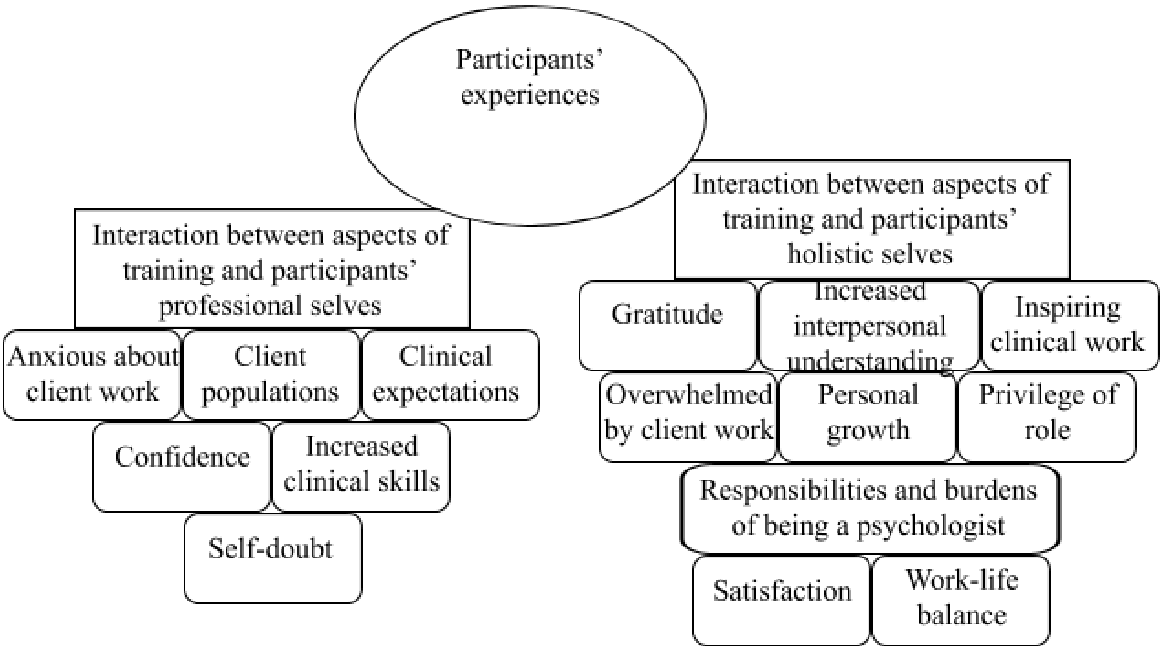

Figure 1. Summary of the sub-themes relating to participants' experiences

The second theme (see Figure 2) was *self-care* and contained four sub-themes including *knowledge*, *practices*, *frequency*, and *barriers*. *Knowledge* and *frequency* were not

included in this results section as they did not fit the purpose of this article. The authors are contactable for access to the full self-care analysis. *Practices* consisted of four further sub-themes: *personal, peer support, supervision,* and *systemic support*. *Barriers* consisted of three further sub-themes: *personal, supervision limitations,* and *systemic limitations*.

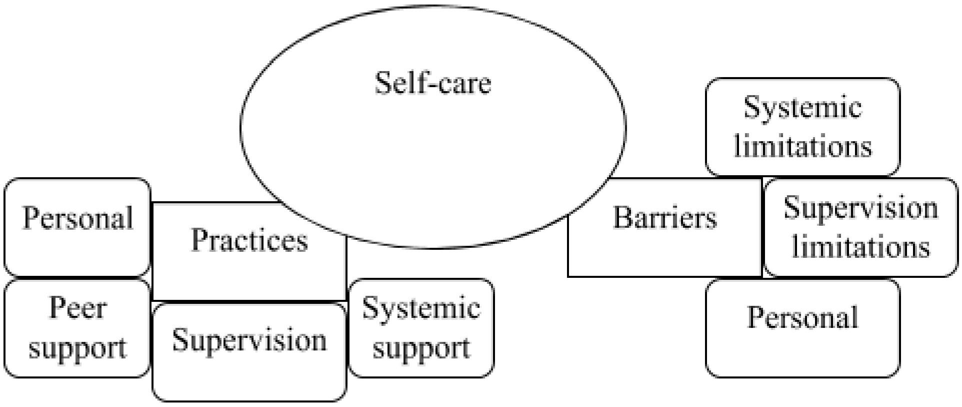

Figure 2. Summary of the sub-themes relating to self-care

Participants' Experiences

Interaction between aspects of training and participants' professional selves.

Anxious about client work. In this general sub-theme, all 14 participants used phrases and words such as "unprepared", "going in blind", "out of my depth", "thrown in the deep end", "no idea what we were doing", "unknowledgeable", and "I felt totally incompetent". These expressions conveyed the feeling of fear or anxiety caused by being uncertain of what to expect and whether they would be able to handle working with clients. Peaks in anxiety occurred whenever a new setting was introduced. An anxiety peak occurred at the beginning of placement one (the first placement, which was at UC's internal clinic), but not at any stage throughout placement two (which was also at UC's internal clinic, meaning that the familiarity of placement one continued). Other peaks in anxiety occurred at the beginning of placement three (the third placement, which was external to UC), anticipating placement four

(the fourth placement, which was at a new location external to UC), and anticipation of entering the workforce.

I felt like I didn't have ... anything to offer and to be sitting there as a psychologist, provisional psychologist ... there's that period of time where you feel like a fraud…

In addition, a typical sub-theme was observed, through eight participants acknowledging that exposure to clients is how a lot of learning eventuated, which increased familiarity and comfort levels, in turn decreasing their feelings of anxiety.

> *…only until you actually start working with clients that you really get a sense of how what you’ve learnt theoretically can be applied ... you’re never going to be trained adequately before you see your first client ... you can’t learn except by doing it ... there’s got to be a space in the beginning where you’re just floundering.*

> *I got to a point where I started feeling like I had some tools or knew how to, at least, do the very basics. And then the anxiety started, sort of, coming down.*

Client populations. Thirteen participants’ reports constitute this general sub-theme related to the challenge of working with one or more client population. The client groups that challenged the greatest number of participants were those with high risk of self-harm/suicide; reported by five participants, and those who had been subjected to abuse or trauma; also reported by five participants.

> *…caring for people’s safety so, like, I’ve had clients who with, like, suicidal ideation and you just - you get very worried about their safety and their wellbeing and if they’re being - going to be taken care of and looked after properly…*

> *…care and protection, um, issues ... if you report, the child might get into a safer environment - if you don’t report, um, they might get hurt but you might maintain a therapeutic relationship…*

...disclosures of sexual abuse, um, seeing how that has affected the person. Um, yeah, it can be - for me I find it - it can be very upsetting witnessing someone else's pain resulting from those things.

Clinical expectations. A variant sub-theme was established through five participants reporting they had held expectations regarding client change. Participants spoke of expectations around being in control and able to affect client change, and what type of change would occur. Discomfort in relation to the concept of "accepting" rather than "changing" client issues was expressed; there was a desire to "fix" client "problems" and fulfil the helper role. Four of these participants spoke of these expectations shifting, through realising, although they could support and have an influence on clients, uncontrollable client factors also determined change.

I had very high expectations of what I would be able to do as a psychologist ... now I realise that my expectation was unrealistic and that it's okay to work towards it but ... it's okay not to be there yet ... [what is important is] understanding them [clients]. Understanding how I am with them ... what they can do, what they can't do. And what I can do for them.

Confidence. This typical sub-theme relates to confidence, with nine participants reporting that factors such as having developed a familiarity with clinical work, seeing change occur in clients, being viewed as a knowledgeable professional worthy of being in control and trusted, and having purpose and something to give. These factors led them to be more comfortable and competent in their clinical work, with an increased ability to problem-solve and deal with the unknown.

...more familiar with what I need to do, more confident in what I'm doing, more, um, and I guess also each time feeling more and more like I have something to offer as I learn ... I feel much more natural in the role now.

Increased clinical skills. A general sub-theme was composed of 13 participants believing their clinical knowledge and skill base had improved. The span of this development covered general abilities such as professionalism, functioning in a workplace, time management, and problem-solving; administrative tasks such as organising appointments, writing session notes and reports, making and returning phone calls, and delivering feedback; and clinical tasks such as researching to prepare for client sessions, conducting initial interviews, making diagnoses, carrying out therapy, assessing risk, establishing and maintaining boundaries, and remaining positive about client outcomes regardless of what the outcome was.

Self-doubt. In relation to their work with clients, six participants had experienced low confidence, a strong self-critical voice, and negative self-evaluation. These experiences formed the variant sub-theme, self-doubt. These data involved participants questioning their overall competency and ability to be a good psychologist, their communication skills, if client changes were naturally occurring or because of therapy, and if they were doing something wrong or if it was about them as a person when client change did not occur or they did not connect with some clients as well as others.

> *...you question yourself more because you say, "Oh am I doing this right? Or is this been helpful?" ... and I guess you question yourself because even yeah do I really want to do this? Do I really want to work in this area ... am I going to be any good at this area...*

Interaction between aspects of training and participants' holistic selves.

Gratitude. A variant sub-theme was established through three participants' reports that exposure to clients had highlighted a sense of gratitude they felt toward aspects of their own lives.

...when you see all these kinds of people experiencing different problems be it you know, emotional, mental health, physical health ... it makes you reflect on your own life ... what good things you have in your own life and that you take for granted sometimes, so your own mental health and your own kind of functioning and, um, life satisfaction ... a stable life where, you know, you have good parents ... you're grateful [for those things].

Increased interpersonal understanding. A typical sub-theme was identified when nine participants reported that exposure to the diversity of clients and client experiences fostered an increase in awareness of other people and what other people go through, awareness of mental health in their own self as well as other people (including family and friends in addition to clients), awareness of pain and suffering, tolerance of pain and suffering, ability to see things from other people's perspectives, ability to apply a non-judgemental attitude to other people and their experiences, and acceptance of diversity.

...they come with their struggles and I see that they're - like I see a sense of humanity there, like we're all people, we all struggle and I see these people coming in with a struggle and like it makes me reflect on my own humanity as a person where I struggle and that we're all - like we all have our struggles.

Inspiring clinical work. Five participants' reports formed the variant sub-theme that conveyed that they found being exposed to clients, the challenges clients had experienced in their lives, and the way clients worked to overcome such challenges, inspiring.

Inspiring is definitely the clients. Absolutely. The clients are amazing. Like the - just the, um, the - the struggles that some people have gone through and you just think, you know, they're working hard, they're health seeking, they're working hard to, um, to overcome challenges. And I find that really inspiring. I've always found that really inspiring ... what they've lived through, it's just incredible.

Overwhelmed by client work. This typical sub-theme was identified when words such as “demanding”, “stressful”, “pressure”, “time consuming”, and “exhausting” were used by 12 participants to describe the experience of client work, including placement attendance, administrative tasks, and clinical tasks; as well as accessing supervision and collaborating with peers who were at times “tired”, “strung out”, and “stressed” because of going through the same overwhelming experience.

> *...you can feel like you're being pushed past what you can provide ... I think that's really challenging 'cause it... you just want to be able to give everything.*

Personal growth. Thirteen participants reported personal growth, which led to this general sub-theme. This growth spanned across individual, interpersonal, and environmental domains. Individual growth included increased self-awareness, ability to manage own experiences, and “groundedness”; having become a stronger person; and increased certainty and understanding of career pathways, personal values, and driving forces. Interpersonal growth included improved communication, increased patience, and increased capacity to understand others and deal with situations involving others. Environmental growth included increased ability to accept things that cannot be changed, maintain openness in response to things “not going one's own way”, “take things as they come”, and respond to things in a calmer manner.

> *...these things that elicit stress are also the most rewarding, once they're done and once you feel like, if that happens again, I know what to do now. I sort of have these tools that I can use to make it better which I previously didn't have so it's almost that catharsis is really beneficial.*

Privilege of role. This typical sub-theme conveys how participants experienced clients coming to them - strangers - for help. What it was like for clients to open up and share very personal and vulnerable parts of themselves. To describe this experience, eight

participants used words such as “humbled”, “privilege”, “touched”, “appreciate”, “take seriously”, and “extraordinary to be trusted”.

…in terms of the value of trust and the - how, um, open and trusting clients are to a psychologist or a mental health professional … the magnitude of that role in someone’s life is, sort of, like, it’s helped me to sort of appreciate and respect the role of a psychologist and that’s not something to be abused and to take lightly and it’s quite an honour.

Responsibilities and burdens of being a psychologist. This variant sub-theme involved six participants experiencing a sense of clients relying on them; wanting to do well in their work with, or believing they were responsible for the outcomes of, clients; and excessively thinking about clients’ situations and questioning whether they had done enough, or the right things, to help. Other burdens participants reported experiencing included having trouble separating their professional and personal identities or ways of being, discomfort with the client-clinician power imbalance, and concern about seeing clients outside of practice.

I think it’s just that like worry of like you’re trying to help people and like if you fail at that… sort of the implications of that. And um yeah just that kind of like responsibility that’s on you to… to be a psychologist and to help other people…

Satisfaction. Twelve participants used words such as “enjoyment”, “accomplishment”, “achievement”, “reward”, “gain”, and “self-pride” to explain how they felt about the processes of learning and trying new things, witnessing client change, and being appreciated for their clinical contribution, despite the associated challenges. These data resulted in the typical sub-theme, satisfaction.

I would like now just to say overall how I feel about my experiences … It's such a, um, kind of rollercoaster, I think I said that before, because it's so awesome and you learn

so much, but it's also so horrible sometimes, and it makes you feel the worst you've ever felt, but also the best you've ever felt. But mostly the best you've ever felt…

Work-life balance. A typical sub-theme was comprised by experiences of difficulty in maintaining a work-life balance, which was reported by eight participants. This difficulty involved balancing activities relating to the degree, paid work, and personal life. Participants described experiences of being "burdened with stress"; as if their lives were "intruded on", "consumed", "fragmented", "siloed", and "not integrated at all" as a result of the degree.

…my life is not my own anymore … it's taken over my whole life … my free time is no longer my free time. I would like to have a better work-life balance than I currently have … I don't know how you get around that.

Self-care

Practices.

Personal. A general theme was established through all 14 participants reporting a variation of personal self-care strategies. The most common form reported was relaxation and pleasure activity, which all 14 participants indicated they engaged in - examples of these activities were watching TV, reading, mindfulness, going to the beach, shopping, video games, and bathing. Twelve participants engaged in exercise, the same number in social contact outside of their family, eight in contact with family, and eight in maintaining their diet. Six participants described activities relating to their own personal mental health care - examples of this being acknowledging limitations such as time and financial pressures, writing in a diary, and applying the therapeutic model "Acceptance and Commitment Therapy" to oneself. Five participants reported maintaining a work/life balance by "leaving work at work" and ensuring they focus on non-work activities. Finally, four participants engaged in maintaining their sleep, and two in spirituality.

Peer support. A typical theme involved twelve participants describing their student cohort as a group of nice people who got along, had fun, and were “close”, “reassuring”, “encouraging”, “comforting”, and “protective of each other”. Participants spoke of how significant the support obtained through this group was, using phrases such as “got me through it”, “kept me going”, “same boat”, and “bring each other up” to describe the experience of going through the program with people at the same level, saying that experiences were normalised and skills were learnt through informally discussing and relating about anxieties, client work, and systemic matters.

> *…we do feel like we’re our own little, kind of family, kind of thing - our little psych family…*
>
> *I don’t think I could have done it without … I would have fallen to pieces if we didn’t have each other.*

Supervision. Clinical supervision appeared a valuable support; a general theme consisted of a report from each of the 14 participants. Participants expressed that the guidance and impact supervisors had on them played a part in them navigating the program. Participants described supportive supervisors as those who had a personable and warm interpersonal nature, were accessible, were confidential, and listened. Furthermore, value was found in supervisors who shared experience and skills to learn from, delivered constructive criticism to push growth, and treated them as equals and fostered autonomy. Supportive supervisors were also described as motivated, successful, and interested to be a part of the process themselves.

> *…having someone who can mentor you, um, is, is absolutely priceless … my supervision sessions aren’t just about clients, they're actually about me as a therapist and me as, as a person and what I bring…*

Systemic support. A typical theme was formed through seven participants reporting positive aspects of the program structure. Notable aspects mentioned were: placements being organised for every student, good experience on placements, opportunity to try different forms of practice through placements - e.g. forensic - to determine career suitability, extra-curricular opportunities which contributed to skillsets, and approachable staff. Being treated like a member of the team instead of a student made a difference, as did monthly drinks with all the staff and students. Regarding coursework, these participants reported that it had been practical, applied, and useful, and that the balance of classes with the rest of the program was right.

> *I feel like - like I'm really glad that I'm in this program and that I'm doing Masters, um, and I'm really happy that I was given the opportunity to be in the program, um, and to study to become a psychologist and like, um, I was always going to do it at this uni - this was always where I wanted to do it.*

Barriers.

Personal. The most common personal barrier to practicing self-care was time limitations, with reports from 11 participants. This typical theme involved participants reporting being short of time due to having to balance the MCP workload - class, assignments, placement, and thesis - with the rest of life.

> *I think the main barrier is just that you get too busy and you tend to forget what you preach.*

Sense of responsibility as a barrier to practicing self-care was a variant theme reported by four participants, which can be summarised as withholding from leisure activities out of guilt.

...because I've got - yeah, other obligations and things that I have to do and - it's ironic, actually, I'm at the point where my self-care is probably causing anxiety because I'm pushing aside maybe things that I should do.

Supervision limitations. A typical theme was identified through 11 participants' reports of supervision limitations. These included a desire for more individual supervision hours each week and more varied and flexible options for group supervision, as well as greater consistency in what supervisors were willing to offer in terms of availability and support.

The staff are asked to do so many things, that their focus really isn't on the students, and the student experience ... we have so many clinicians and people who are a part of the degree that have such valuable experience and we can't actually access that because there's no capacity for them to provide that.

Systemic limitations. This typical theme was developed through data from 10 participants. Participants' reports included challenges related to the structural organisation of the course, a desire for clearer communication between staff and students, greater practical application in coursework units, and wanting options to indicate external placement preferences. Participants also reported limited access to computers and free printing of clinical material, and feeling a lack of support with respect to implementing self-care.

...overall for self-care I question it a bit ... with such a kind of high stress, high workload kind of course ... I think it's important how the university kind of actually takes care of its students ... they encourage a lot of self-care but ... what they can control they don't do much about ... [the structure of the program could] enhance skills without overloading them [students] with too much...
